# DcR3 Suppresses Lipopolysaccharide-Induced Aggresome-like Structures in Macrophages via Inhibition of Reactive Oxygen Species and p38 MAPK

**DOI:** 10.3390/ijms27146433

**Published:** 2026-07-20

**Authors:** Chun-Hung Lee, Duen-Yi Huang, Shie-Liang Hsieh, Yuan-Shen Chen, Wan-Wan Lin

**Affiliations:** 1 Department of Pharmacology, College of Medicine, National Taiwan University, Taipei 10051, Taiwan; 2 Institute of Clinical Medicine, College of Medicine, National Yang Ming Chiao Tung University, Taipei 112304, Taiwan; 3 Immunology Research Center, National Health Research Institutes, Zhunan 35053, Taiwan; 4 Department of Medical Research and Education, Taipei Veterans General Hospital, Taipei 112201, Taiwan; 5 Department of Microbiology and Immunology, College of Medicine, National Yang Ming Chiao Tung University, Taipei 112304, Taiwan; 6 Department of Neurosurgery, National Taiwan University Hospital Yunlin Branch, Douliu 64041, Taiwan

**Keywords:** DcR3, ALIS, ubiquitination, lipopolysaccharide, bone marrow-derived macrophages, inflammation, autophagy

## Abstract

Decoy receptor 3 (DcR3) is a pleiotropic soluble factor that modulates cellular functions through both decoy and non-decoy mechanisms. DcR3 has been reported to exert anti-apoptotic and anti-inflammatory effects in humans, particularly in cancers and inflammatory diseases. In the present study, we investigated the role of DcR3 in TLR4-mediated innate immune responses in macrophages. Because the *DcR3* gene is absent in the mouse genome, we generated myeloid-specific DcR3 knock-in mice and isolated bone marrow-derived macrophages (BMDMs) for functional analyses. Our results showed that DcR3 did not significantly affect LPS-induced expression of COX-2, iNOS, NLRP3, or pro-IL-1β. Aggresome-like induced structures (ALIS), which consist of aggregates of ubiquitinated proteins, are stress-induced cytoplasmic compartments implicated in MHC class I antigen presentation. We found that DcR3 suppressed LPS-induced ALIS formation by attenuating cellular reactive oxygen species production and p38 MAPK activation. In addition to LPS stimulation, DcR3 also reduced the accumulation of ubiquitinated proteins induced by HO-1 inhibitor ZnPP, lysosomal inhibitor bafilomycin A1, and proteasomal inhibitor MG132. Consistent with a role for autophagy in ALIS regulation, rapamycin reduced LPS-induced ALIS formation, whereas bafilomycin A1 induced comparable LC3-II accumulation in both wild-type and DcR3-expressing macrophages. Furthermore, DcR3 expression did not significantly alter LPS-induced p62 or HO-1 expression. Collectively, although DcR3 does not markedly influence LPS-induced inflammatory responses in BMDMs, our findings reveal a previously unrecognized role for DcR3 in suppressing ALIS formation and the accumulation of ubiquitinated proteins in macrophages, thereby suggesting a novel function for DcR3 in maintaining intracellular protein homeostasis under stress conditions.

## 1. Introduction

Decoy receptor 3 (DcR3), also known as tumor necrosis factor receptor superfamily 6b (TNFRSF6B or TR6), is a member of the tumor necrosis factor receptor (TNFR) superfamily. Unlike most members of the TNFR superfamily, DcR3 lacks a transmembrane domain and is detectable in serum and cell culture medium. DcR3 comprises a signal peptide, four cysteine-rich domains (CRDs), and a heparan sulfate proteoglycan (HSPG) binding domain (HBD) [1,2]. DcR3 is present in human and chimpanzee genomes but absent from mouse and rat genomes. This soluble receptor is almost undetectable in non-pathologic conditions but may play an important role in immune modulation and cancer progression. Previous studies showed the high expression of DcR3 in patients with a variety of cancers and inflammatory conditions, including sepsis, acute respiratory distress syndrome, Crohn’s disease, kidney disease, hepatitis, and rheumatoid arthritis [3,4,5,6,7,8].

DcR3 is an effector in promoting cancer development. Many types of tumors express high levels of DcR3, enabling cancer cells to escape immune surveillance and promoting angiogenesis. These actions of DcR3 are primarily via its neutralizing and decoy actions on Fas ligand (FasL), LIGHT, and TL1A [3,9,10]. FasL is a death-inducing ligand that activates the extrinsic apoptotic pathway mediated by Fas and acts as a tumor suppressive mechanism [11,12]. LIGHT generally enhances antitumor immunity by binding to herpesvirus entry mediator (HVEM) and lymphotoxin β receptor (LTβR), thereby stimulating T-cell activation, dendritic cell maturation, and cytotoxic immune responses within the tumor microenvironment [13]. TL1A interacts primarily with death receptor 3 (DR3) and can enhance antitumor immunity by promoting activation of effector T cells and natural killer cells, as well as suppressing angiogenic activity in some tumor settings [14,15].

In addition to its neutralizing effect, DcR3-mediated effector function (also known as non-decoy function) modulates immune responses and is implicated in inflammatory diseases [3,4,8,16]. The non-decoy effector function of DcR3 is primarily based on the action via its HBD, which comprises a stretch of positive amino acid residues (aa 256-261) located outside the ligand-binding region, to activate HSPGs, such as syndecan 2 and CD44v3 [17]. Moreover, the exogenous HBD.Fc-mediated effect is independent of the neutralizing actions and is regarded as non-decoy actions of DcR3 [18]. Studies using the DcR3-Fc fusion protein (DcR3.Fc) further reveal multiple non-decoy anti-inflammatory functions of DcR3, highlighting its promising therapeutic potential [7,19]. For example, DcR3 can reduce aberrant protein structures in the brain, which is related to the pathogenesis of Alzheimer’s disease [20]. DcR3.Fc skews T lymphocytes to Th2 cells and shows a Th1-suppressive effect [21]. DcR3.Fc-treated macrophages exhibit M2 polarization and suppress phagocytic activity [20,22]. DcR3.Fc-differentiated macrophages display a reduction in influenza A virus-induced pulmonary inflammation [18]. Moreover, DcR3 was shown to reduce NLRP3 inflammasome activation in lipopolysaccharide (LPS)-primed macrophages, and this effect is related to the reduction in reactive oxygen species (ROS) production and lysosomal rupture [23]. In disease models, DcR3.Fc can suppress cecal ligation and puncture (CLP)-induced sepsis [16], kidney transplant rejection [24], but enhance functional recovery after spinal cord injury [25]. Regarding antigen presentation and antibody formation, DcR3-Fc attenuates T cell-dependent antibody production via attenuating proliferation, NF-κB activity, and Xbp1 expression in Blimp1^+^ antibody-secreting cells [26,27]. Such a suppression effect of DcR3.Fc in B cell functions has a negative correlation with disease activity in rheumatoid arthritis [28]. In addition, the expression of genes that are involved in major histocompatibility complex (MHC)-II-dependent antigen presentation, along with class II transactivator (CIITA), is suppressed by DcR3.Fc possibly through epigenetic modification [29].

Aggresome-like induced structures (ALIS) are a specific cellular stress response [30,31] and are initially observed in LPS-treated dendritic cells, characterized by circular aggregates of ubiquitinated proteins [32]. The cellular functions of ALIS include temporary storage of misfolded and ubiquitinated proteins, regulation of innate immune responses, and antigen processing and presentation [31,33]. LPS stimulation markedly increases protein synthesis, ROS generation, and inflammatory signaling, creating a burden on protein quality-control systems. Under these conditions, misfolded and ubiquitinated proteins accumulate and are sequestered into ALIS. Several studies suggest that ALIS represent a coordinated adaptation during macrophage activation, allowing cells to maintain proteostasis while sustaining inflammatory responses. ALIS is also considered an antigen storage compartment, relating to antigen presentation with an increasing number of MHC class I molecules and costimulatory molecules [34]. Studies demonstrate that many kinds of stresses, such as LPS, starvation, heat shock, catalase inhibition, proteasome inhibition, infection, and ER stress, can induce ALIS formation [31,35,36,37]. Moreover, TLR-mediated ALIS comprises antimicrobial peptides to attenuate intracellular bacterial survival [38]. Studies further demonstrate ALIS acting as microdomains sensing cellular stresses for triggering oxidative stress-induced parthanatos [39,40,41].

Both the autophagosome–lysosome pathway and ubiquitin–proteasome system are involved in the clearance of ALIS [31,36,42]. In response to oxidative stress, proteotoxic stress, or microbial infection, K48-linked polyubiquitinated proteins within ALIS are recognized by selective autophagy receptors such as p62/SQSTM1, which facilitate their sequestration into autophagosomes for lysosomal degradation [37,40,41,43,44,45,46]. In addition, LC3, an autophagy-related protein that interacts with p62, is colocalized with ALIS [44,45]. In addition to autophagy, the ubiquitin–proteasome system also contributes to ALIS turnover by refolding or degrading misfolded proteins before aggregate accumulation [31,36]. Through coordinated proteostasis and autophagic mechanisms, ALIS clearance helps restore cellular homeostasis and prevent excessive inflammatory or proteotoxic stress.

Although DcR3 is known to exert anti-inflammatory and immunomodulatory functions and regulate cellular stress responses, its role in intracellular protein quality-control pathways remains largely unexplored. Therefore, investigating the involvement of DcR3 in LPS-induced ALIS formation may reveal a novel function for DcR3 in maintaining proteostasis and mitigating cellular stress during innate immune responses. Because the DcR3 gene is absent from the mouse genome, we generated DcR3 transgenic (Tg) mice with high DcR3 expression. Indeed, DcR3 transgenic (DcR3 Tg) mice and isolated bone marrow-derived macrophages (BMDMs) from DcR3 Tg mice have been used to show the multi-functions of macrophage-specific DcR3 [8,20,23,24,47]. In this study, our finding that DcR3 suppresses ALIS formation suggests a previously unrecognized role for DcR3 in limiting proteotoxic stress and preserving intracellular protein homeostasis during inflammatory activation.

## 2. Results

### 2.1. DcR3 Does Not Alter LPS-Induced Inflammatory Responses in BMDMs

**Figure 1:**
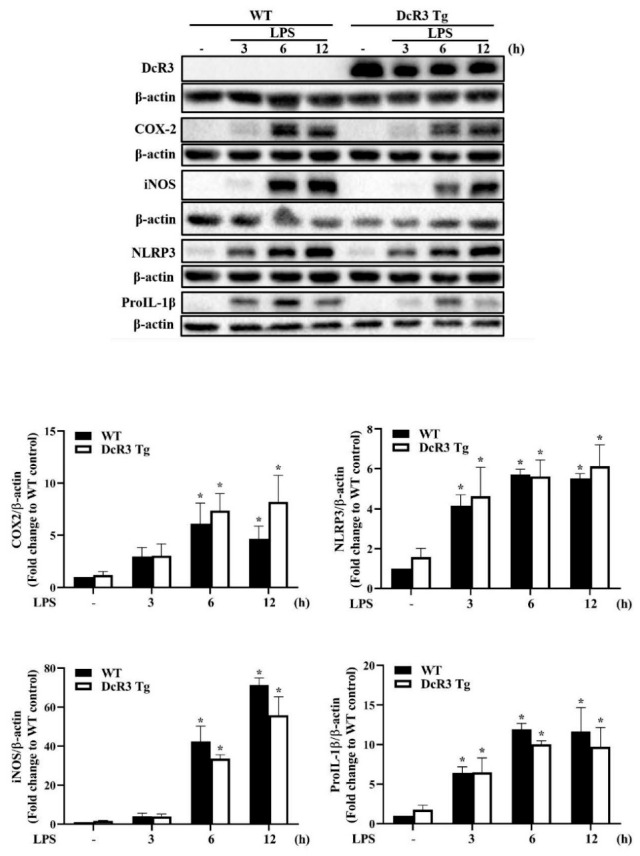
DcR3 does not alter LPS-induced inflammatory responses in BMDMs. WT and DcR3 Tg BMDMs were treated with LPS (100 ng/mL) for the indicated times. Total cell lysates were collected and analyzed by immunoblotting with specific antibodies for COX-2, iNOS, NLRP3, and proIL-1β. Quantitative analyses were normalized with β-actin and expressed as relative fold change to the WT control group. Data were presented as the mean ± S.E.M from at least three independent experiments. * *p* < 0.05, indicating a significant increase in LPS-induced inflammatory responses in WT and DcR3 Tg BMDMs.

To investigate the role of DcR3 in TLR4-mediated inflammatory responses, we used BMDMs stimulated with LPS as an experimental model. Because the DcR3 gene is absent from the mouse genome, BMDMs were isolated from myeloid-specific DcR3 Tg mice for functional analyses. In wild-type (WT) BMDMs, LPS treatment induced a time-dependent increase in the protein expression of COX-2, iNOS, NLRP3, and proIL-1β. However, the expression levels of these inflammatory mediators were comparable between DcR3 Tg and WT BMDMs following LPS stimulation (Figure 1). In addition, LPS-induced IL-6 gene expression was not changed by DcR3. These findings suggest that DcR3 does not significantly modulate LPS-induced inflammatory responses in BMDMs.

### 2.2. DcR3 Decreases LPS-Induced ALIS Formation

**Figure 2:**
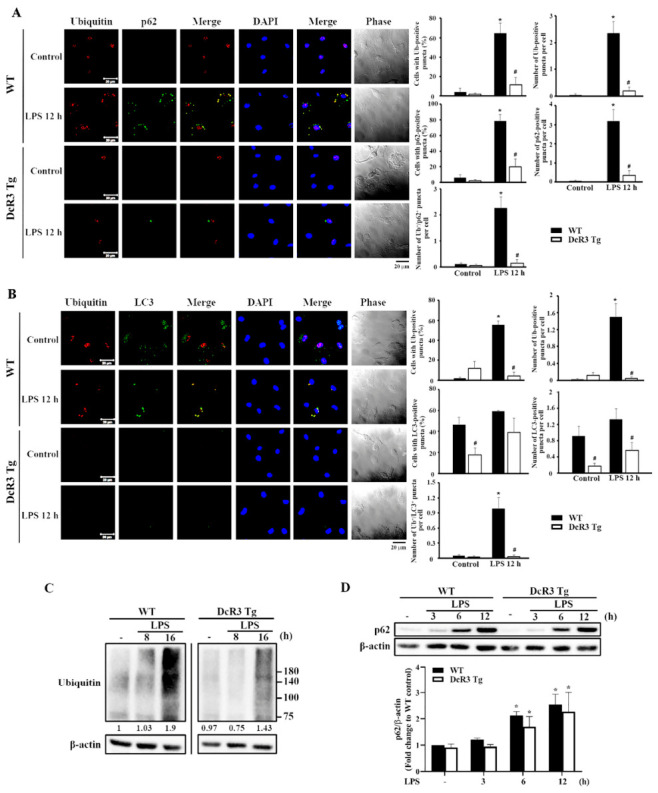
DcR3 expression reduces ubiquitinated protein and ALIS puncta in BMDMs after LPS stimulation. (**A**,**B**) Representative confocal images of WT and DcR3 Tg BMDMs treated with LPS (100 ng/mL) for 12 h. Immunofluorescence staining was performed to detect ubiquitin (red color), p62 (green color) (**A**), and LC3 (green color) (**B**) expression and their co-localization. Nuclei were counterstained with DAPI (blue color). Scale bar: 20 μm. The percentages of cells with specific puncta and puncta number per cell were quantified. (**C**,**D**) WT and DcR3 Tg BMDMs were treated with LPS (100 ng/mL) for the indicated times. Total cell lysates were collected and analyzed by immunoblotting to determine ubiquitinated proteins (**C**) and p62 (**D**). β-Actin was detected as a loading control. The expressional changes were determined after normalization with β-action and presented as fold change compared to basal response in the control cells. Data were presented as the mean ± S.E.M from at least three independent experiments. * *p* < 0.05, indicating a significant increase in LPS-induced ubiquitin (**A**,**B**), p62 (**A**), and LC3 punctate (**B**), as well as p62 expression (**D**) in WT and DcR3 Tg BMDMs. # *p* < 0.05, indicating a significant inhibitory effect of DcR3 on LPS-induced punctate of ubiquitin, p62 and LC3.

Although DcR3 did not significantly affect LPS-induced expression of inflammatory mediators, we explored the possibility that DcR3 may regulate LPS-induced ALIS formation. To identify ALIS, WT BMDMs were stimulated with LPS and stained with an anti-ubiquitin antibody. Confocal microscopy revealed the formation of prominent cytoplasmic ubiquitin-, p62-, and LC3-positive puncta following LPS stimulation in WT cells. Moreover, ubiquitin-positive puncta were colocalized with either p62- (Figure 2A) or LC3 (Figure 2B), consistent with the characteristics of ALIS reported previously [44,45]. These effects of LPS, presented as percentages of cells with Ub^+^ or p62^+^ puncta, Ub^+^ or p62^+^ puncta number per cell, and Ub^+^/p62^+^ double-positive puncta number per cell, were markedly attenuated by DcR3 (Figure 2A). Because after LPS stimulation the percentage of Ub-positive cells was similar to that of either p62 or LC3, in the following experiments we used Ub staining as an index of ALIS puncta.

We next examined the accumulation of ubiquitinated proteins by immunoblot analysis. Consistent with the confocal microscopy results, the characteristic ubiquitin smear was substantially reduced in DcR3 Tg BMDMs compared with WT cells (Figure 2C), indicating decreased accumulation of ubiquitinated proteins. Because p62 plays an essential role in LPS-induced ALIS formation [45], we further assessed p62 protein expression. As expected, LPS induced a time-dependent increase in p62 expression; however, no significant difference in p62 protein levels was observed between WT and DcR3 Tg BMDMs throughout the treatment period (Figure 2D).

### 2.3. ALIS Formation Requires ROS Production and p38 Activation, and DcR3 Exerts an Inhibition

**Figure 3:**
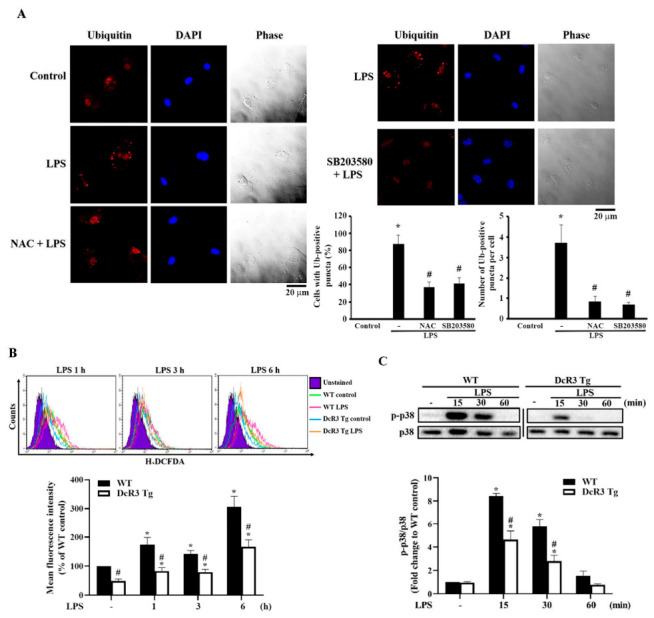
LPS-induced ROS production and p38 activation contribute to ALIS formation and DcR3 attenuates both events. (**A**) Representative confocal images of WT BMDMs treated with LPS (100 ng/mL) for 12 h in the absence or presence of pretreatment with NAC (5 mM) or SB203580 (0.5 μM) for 30 min. Immunofluorescence analysis was performed to detect ubiquitin. Nuclei were counterstained with DAPI. The percentages of cells with Ub puncta were quantified. (**B**,**C**) WT and DcR3 Tg BMDMs were treated with LPS (100 ng/mL) for the indicated times. Cellular ROS level was determined by H_2_DCFDA (10 μM) (**B**). The fluorescence intensity of the WT control group was set as 100% to present relative values. Data were presented as the mean ± S.E.M from at least three independent experiments. * *p* < 0.05, indicating a significant increase in LPS-induced cellular ROS in WT and DcR3 Tg BMDMs. # *p* < 0.05, indicating significant attenuation of cellular ROS in DcR3 Tg BMDMs compared to WT groups. Total cell lysates were collected and analyzed by immunoblotting to determine protein levels of phosphorylated p38 and total p38 (**C**). Quantitative analysis of phosphorylated p38 was normalized with total p38, and relative fold change to the WT control group was presented. Data were presented as the mean ± S.E.M from at least three independent experiments. * *p* < 0.05, indicating significant effects of LPS-induced cellular ROS production and p38 activation in WT and DcR3 Tg BMDMs. # *p* < 0.05, indicating significant attenuation of LPS responses in DcR3 Tg BMDMs compared to WT groups.

Next, we sought to elucidate the mechanism by which DcR3 suppresses ALIS formation. Previous studies have demonstrated that ROS generation and p38 MAPK activation downstream of TLR4 signaling contribute to ALIS formation in macrophages [45]. To confirm their involvement in our experimental system, WT BMDMs were pretreated with N-acetyl-L-cysteine (NAC), a ROS scavenger, or SB203580, a selective p38 inhibitor, before LPS stimulation. Consistent with previous reports, pretreatment with either NAC or SB203580 markedly reduced LPS-induced ubiquitin-positive puncta, indicating suppression of ALIS formation (Figure 3A). We next examined whether DcR3 modulates ROS production in BMDMs. Intracellular ROS levels were measured using H2DCFDA, and the results showed that DcR3 expression significantly reduced cellular ROS levels under both basal and LPS-stimulated conditions (Figure 3B). In parallel, immunoblot analysis revealed that LPS-induced p38 MAPK phosphorylation was attenuated in DcR3-expressing BMDMs compared with WT cells (Figure 3C). Taken together, these findings suggest that DcR3 suppresses LPS-induced ALIS formation, at least in part, through the inhibition of ROS generation and p38 MAPK activation.

### 2.4. DcR3 Does Not Alter LPS-Induced HO-1 Expression

**Figure 4:**
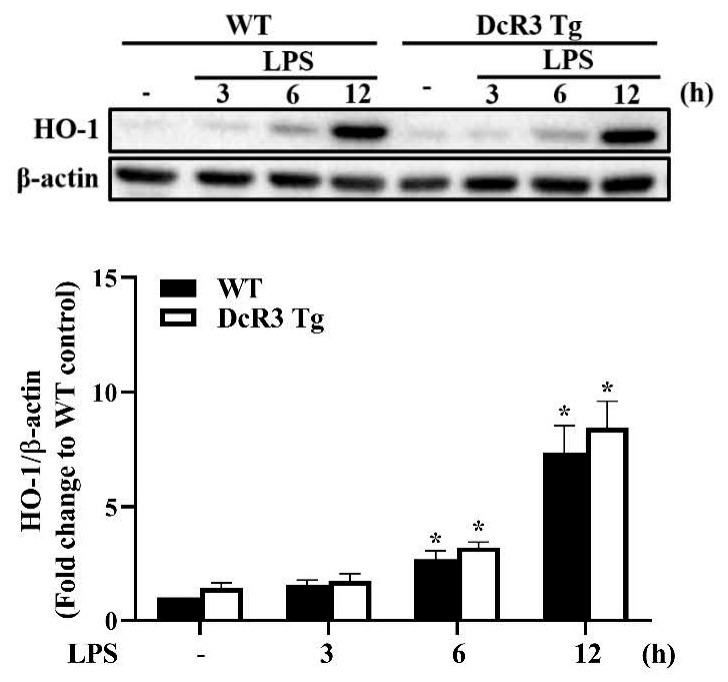
DcR3 does not alter LPS-induced HO-1 protein expression. WT and DcR3 Tg BMDMs were treated with LPS (100 ng/mL) for the indicated times. HO-1 protein was analyzed by immunoblotting. Data were presented as the mean ± S.E.M from at least three independent experiments. * *p* < 0.05, indicating significant increase in HO-1 expression in WT and DcR3 Tg BMDMs.

Heme oxygenase (HO-1) is a stress-inducible enzyme that catalyzes the degradation of heme. In addition to its cytoprotective role against various cellular stresses, including LPS exposure, hypoxia, hyperthermia, and irradiation, HO-1 also exerts potent anti-inflammatory and antioxidant effects [48]. Interestingly, both HO-1 and DcR3 have been reported to protect pancreatic islet cells from apoptosis and improve islet function following allogeneic transplantation [49,50]. Because LPS is known to induce HO-1 expression in macrophages [51,52], we investigated whether DcR3-mediated suppression of ROS production might be associated with enhanced HO-1 expression. Consistent with previous reports, LPS stimulation induced HO-1 expression in WT BMDMs. However, the level of HO-1 induction was comparable between WT and DcR3 Tg BMDMs (Figure 4), indicating that DcR3 expression does not significantly affect LPS-induced HO-1 expression. These results suggest that the inhibitory effect of DcR3 on cellular ROS production is unlikely to be mediated through the upregulation of HO-1.

### 2.5. Hemin and ZnPP Do Not Affect LPS-Induced ALIS Formation in BMDMs, While ZnPP-Induced Ubiquitinated Protein Expression Is Blocked by DcR3

**Figure 5:**
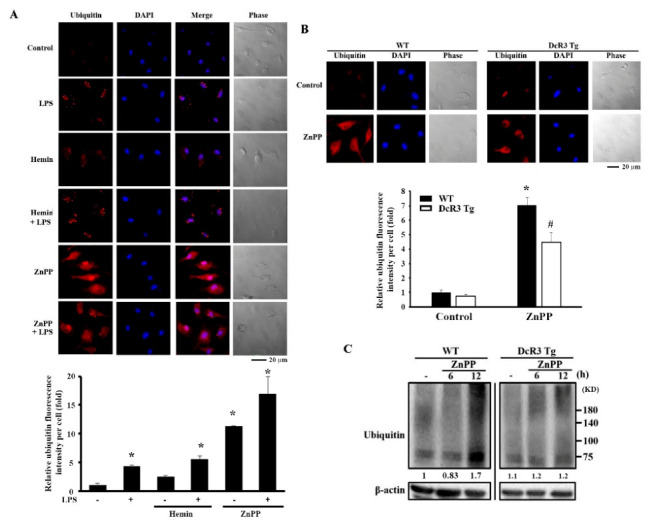
Hemin and ZnPP do not affect ALIS formation in LPS-stimulated BMDMs, and ZnPP-induced ubiquitinated protein expression is blocked by DcR3. (**A**,**B**) Representative confocal images of WT BMDMs pretreated with hemin (40 μM) and ZnPP (10 μM) alone or in combination with LPS (100 ng/mL) as indicated for 12 h. Cells were processed with ubiquitin antibody. Nuclei were counterstained with DAPI. The relative Ub fluorescence intensity in each experimental group were quantified. (**C**) WT and DcR3 Tg BMDMs were treated with ZnPP (10 μM) for the indicated times. Ubiquitinated proteins were analyzed by immunoblotting, and their expressional changes were determined after normalization with β-action and presented as fold change compared to basal response in the control cells. * *p* < 0.05, indicating significant effects of LPS or ZnPP-induced ubiquitinated protein expression in WT and DcR3 Tg BMDMs. # *p* < 0.05, indicating significant attenuation in DcR3 Tg BMDMs compared to WT groups.

Although DcR3-mediated suppression of ROS production was not associated with HO-1 expression, we were interested in determining whether HO-1 activity itself influences ALIS formation. Previously, HO-1-dependent regulation of proteostasis has been demonstrated. To address this question, we examined the effects of hemin, an inducer of HO-1, and zinc protoporphyrin (ZnPP), an inhibitor of HO-1 activity, on LPS-induced ALIS formation. Treatment with hemin (40 μM) alone slightly induced an increase in ubiquitin-positive puncta; however, it did not significantly affect ALIS formation in LPS-stimulated BMDMs (Figure 5A). In contrast, treatment with ZnPP (10 μM) unexpectedly caused a marked accumulation of ubiquitinated proteins in both the nucleus and cytoplasm (Figure 5A). Upon co-treatment with LPS, ubiquitin-positive puncta remained highly abundant, indicating sustained protein aggregation; nevertheless, their formation was markedly reduced in DcR3 Tg BMDMs compared with WT cells (Figure 5B). Consistent with the confocal microscopy findings, immunoblot analysis demonstrated that ZnPP markedly increased the accumulation of ubiquitinated proteins. Notably, this effect was substantially attenuated in DcR3 Tg BMDMs (Figure 5C). These results suggest that DcR3 suppresses the accumulation of ubiquitinated proteins under conditions of impaired HO-1 activity and that the inhibitory effect of DcR3 on protein aggregation is independent of HO-1 induction.

### 2.6. Bafilomycin A1- and MG132-Induced Ubiquitinated Protein Expression Are Blocked by DcR3

**Figure 6:**
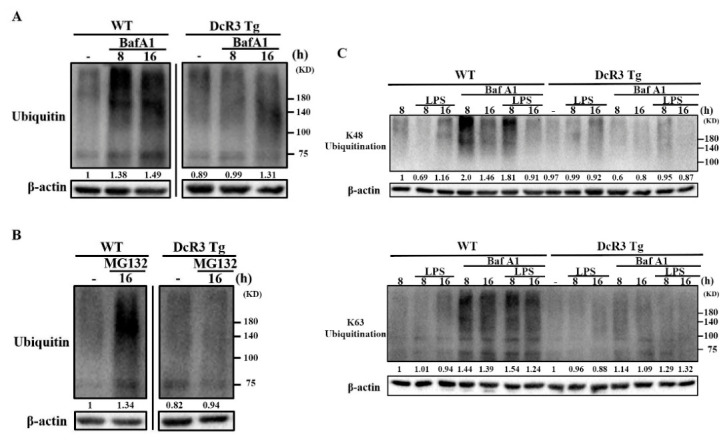
Bafilomycin A1- and MG132-induced ubiquitinated protein expression are blocked by DcR3. WT and DcR3 Tg BMDMs were treated with (**A**,**C**) bafilomycin A1 (100 nM), (**B**) MG132 (10 μM), and (**C**) LPS (100 ng/mL) for the indicated times. Ubiquitinated proteins were analyzed by immunoblotting, and their expressional changes were determined after normalization with β-action and presented as fold change compared to basal response in the control cells.

Given that DcR3 Tg BMDMs exhibited reduced LPS-induced ALIS formation and attenuated ZnPP-induced accumulation of ubiquitinated proteins, we next investigated whether the effect of DcR3 on ubiquitinated protein accumulation could be extended to other proteostasis-disrupting stimuli. Previous studies have demonstrated that ALIS are primarily cleared through two major protein degradation pathways: autophagy and the ubiquitin–proteasome system. Therefore, we examined the effects of bafilomycin A1, an inhibitor of autophagosome–lysosome fusion, and MG132, a proteasome inhibitor, on the accumulation of ubiquitinated proteins. As expected, treatment with either bafilomycin A1 or MG132 markedly increased the total level of ubiquitinated proteins in WT BMDMs. Notably, this accumulation was substantially attenuated in DcR3 Tg BMDMs (Figure 6A,B). Not only total ubiquitinated proteins at the resting state, but we also determined K48 and K63 ubiquitinated proteins upon LPS stimulation. We found that K48- and K63-ubiquitinated proteins that accumulated upon bafilomycin A1 and/or LPS treatment were consistently attenuated in DcR3 Tg BMDMS (Figure 6C). These findings suggest that DcR3 broadly suppresses the buildup of ubiquitinated proteins induced by distinct forms of proteostatic stress.

### 2.7. Autophagy Regulates LPS-Induced ALIS Formation in Macrophages, and DcR3 Does Not Affect Bafilomycin A1-Induced LC3-II Accumulation

**Figure 7:**
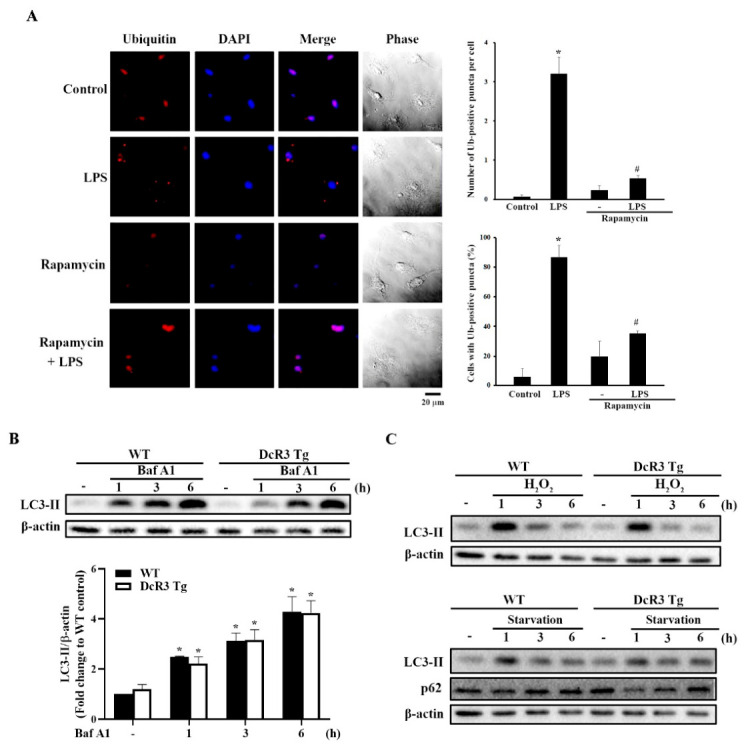
Rapamycin inhibits LPS-induced ALIS formation in macrophages, while DcR3 does not affect bafilomycin A1-induced LC3-II accumulation. (**A**) Representative confocal images of WT BMDMs pretreated with rapamycin (100 nM) alone or in combination with LPS (100 ng/mL). Immunofluorescence staining was performed to detect ubiquitin. The percentages of cells with Ub puncta in each experimental group were quantified. (**B**) WT and DcR3 Tg BMDMs were treated with bafilomycin A1 (100 nM). (**C**) WT and DcR3 Tg BMDMs were treated with H_2_O_2_ (200 μM) or subjected to serum-free culture medium. LC3-II expression was analyzed by immunoblotting. Quantitative analysis of LC3-II was normalized with β-actin and expressed as relative fold change to the WT control group. * *p* < 0.05, indicating significant increases in Ub puncta (**A**) and LC3-II accumulation (**B**) inWT and/or DcR3 Tg BMDMs. #, *p* < 0.05, indicating a significant inhibition of LPS-induced Ub puncta by rapamycin (**A**).

Previous studies have shown that autophagy can be induced in response to oxidative stress. Given our findings that DcR3 attenuates LPS-induced ROS production and that autophagy plays an important role in ALIS turnover, we next investigated whether DcR3 influences autophagic activity in macrophages. To this end, we first examined the effect of rapamycin, a well-established inducer of autophagy through inhibition of mTORC1. The images from confocal microscopy revealed that rapamycin treatment alone had little effect on basal ALIS formation. However, pretreatment with rapamycin markedly reduced ALIS formation in LPS-stimulated BMDMs (Figure 7A), further supporting a role for autophagy in the regulation and clearance of LPS-induced ALIS in macrophages.

Next, we determined whether DcR3 affects autophagic flux. Autophagic flux was assessed by measuring LC3-II accumulation following bafilomycin A1 treatment. During autophagy, cytosolic LC3-I is conjugated to phosphatidylethanolamine and converted into membrane-bound LC3-II, which is incorporated into autophagosomes and subsequently degraded after autophagosome–lysosome fusion. Because bafilomycin A1 blocks autophagosome–lysosome fusion and lysosomal degradation, the resulting accumulation of LC3-II serves as an indicator of autophagic flux. As expected, bafilomycin A1 treatment resulted in the accumulation of LC3-II in WT BMDMs. However, no significant difference in LC3-II levels was observed between WT and DcR3 Tg BMDMs following bafilomycin A1 treatment (Figure 7B). These results suggest that DcR3 does not significantly affect basal autophagic flux under resting conditions. To further clarify the role of DcR3 in stress-induced autophagy, we treated cells with H_2_O_2_ and in serum-free conditions. We found that LC3-II accumulation following both stress conditions was not altered by DcR3 (Figure 7C), strengthening no effect of DcR3 in autophagic flux.

## 3. Discussion

DcR3, originally identified in human cancer cells, is a multifunctional immunomodulatory molecule with anti-apoptotic and anti-inflammatory properties. Previous studies have shown that DcR3.Fc treatment attenuates inflammatory responses in CLP-induced sepsis [16,53], influenza A virus-induced pulmonary inflammation [18], and autoimmune diabetes [54]. In addition, DcR3 expression is upregulated in monocytes and human umbilical vein endothelial cells following TLR2 and TLR4 stimulation [55,56], while DcR3.Fc treatment suppresses the expression of TLR3 and TLR7 in macrophages [18]. Despite these observations, the role of DcR3 in TLR4-mediated inflammatory responses remains poorly understood. Because DcR3.Fc-differentiated macrophages exhibit an M2-like phenotype [20,22], we initially hypothesized that DcR3 would suppress LPS-induced inflammatory responses. To test this hypothesis, we employed BMDMs isolated from DcR3 Tg mice, as the DcR3 gene is absent from the mouse genome. Unexpectedly, we found that LPS stimulation induced comparable levels of COX-2, iNOS, NLRP3, and pro-IL-1β expression in both WT and DcR3 Tg BMDMs. These findings are partially consistent with our previous study, in which no significant differences in LPS-induced NLRP3 and pro-IL-1β expression were observed between DcR3.Fc-differentiated and control human or mouse macrophages [23]. Although numerous studies have demonstrated the anti-inflammatory effects of DcR3, the experimental settings employed in those studies involved more complex inflammatory environments and were not limited to TLR4 signaling alone. For instance, the CLP model induces polymicrobial sepsis, in which inflammatory responses are elicited by a diverse array of Gram-positive and Gram-negative bacteria present in the cecum [57]. Likewise, influenza virus infection activates innate immunity through multiple pattern-recognition receptors, including TLR3, TLR7, TLR8, RIG-I, and NLRP3 [58]. Moreover, the anti-inflammatory effect of DcR3 might be ascribed to its ability to drive macrophages to M2 phenotypes [59,60]. In addition, although exogenous DcR3 can inhibit LPS-induced TNF-α, IL-6, and IL-1β mRNA levels in RAW264.7 macrophages [16], our current experimental condition of endogenously increased DcR3 expression in BMDMs does not significantly affect the rapid expression of major inflammatory mediators downstream of TLR4 activation, suggesting that TLR4 signaling itself is unlikely to be a primary and direct target through which DcR3 exerts its anti-inflammatory effects. Supporting this context-dependent notion, previously in an osteoclastogenesis study in monocyte/macrophage lineage precursor cells, we found that DcR3 itself can induce TNF-α production, even in the absence of LPS stimulation [10].

Since DcR3 did not significantly alter the expression of LPS-induced inflammatory mediators, we next examined other cellular responses elicited by LPS stimulation. ALIS are stress-responsive cytoplasmic structures composed of aggregates of ubiquitinated proteins that have been identified in both professional and non-professional APCs. ALIS formation is closely linked to TLR activation and is thought to serve as a transient storage compartment for antigenic proteins, thereby contributing to MHC class I antigen presentation. Interestingly, previous studies have shown that DcR3 ameliorates amyloid plaque deposition, which is characterized by the accumulation of misfolded Aβ protein aggregates, through the promotion of an IL-4^+^YM1^+^ M2a-like microglial phenotype [20]. This observation led us to hypothesize that DcR3 might also influence the handling of ubiquitinated protein aggregates during macrophage activation. Accordingly, we investigated whether DcR3 regulates LPS-induced ALIS formation. Our results demonstrated that DcR3 markedly suppressed LPS-induced ALIS formation in BMDMs. Consistent with this finding, immunoblot analysis revealed that DcR3 Tg BMDMs accumulate substantially lower levels of ubiquitinated proteins than WT BMDMs following LPS stimulation. These data suggest that DcR3 may participate in regulating protein aggregation and intracellular proteostasis during innate immune activation.

Given the marked reduction in LPS-induced ALIS formation observed in DcR3 Tg BMDMs, we next sought to elucidate the underlying molecular mechanisms. Previous studies have demonstrated that ROS contribute to both LPS- and heme-induced ALIS formation [45,61]. Consistent with this notion, we found that DcR3 attenuates LPS-induced ROS production in BMDMs. Interestingly, DcR3 also reduces basal ROS levels in unstimulated cells, suggesting that DcR3 may influence cellular redox homeostasis under both resting and inflammatory conditions. Cellular ROS are generated primarily from two major sources: NADPH oxidases (NOXs) and the mitochondrial electron transport chain. In macrophages, accumulating evidence indicates that NOXs, rather than mitochondria, represent the predominant source of ROS following LPS stimulation [62,63]. Among the NOX family members, NOX2 is highly expressed in phagocytic cells and plays a central role in antimicrobial defense and oxidative burst responses [64]. Intriguingly, previous studies have shown that exogenous DcR3.Fc suppresses the phagocytic uptake of immune complexes and apoptotic cells by human primary macrophages [22], although it does not affect monosodium urate crystal uptake in PMA-differentiated THP-1 cells [23]. In contrast, DcR3 Tg mice exhibit enhanced uptake of Aβ by microglia [20], suggesting that the effects of DcR3 on phagocytic function may be context-dependent. Although the precise mechanism by which DcR3 reduces ROS production remains unclear, one possibility is that the lower basal ROS levels observed in DcR3 Tg BMDMs are associated with altered NOX2 activity and/or expressional regulation. However, cellular redox status is determined not only by ROS generation but also by antioxidant defense systems, including superoxide dismutases, catalase, glutathione peroxidases, and glutathione reductase [64]. Therefore, further studies are required to determine whether DcR3 regulates ROS homeostasis through modulation of NOX activity, antioxidant capacity, or both.

We next investigated additional signaling pathways that may contribute to the inhibitory effect of DcR3 on ALIS formation. Previous studies have demonstrated that the ROS–p38 MAPK signaling axis plays an important role in LPS-induced ALIS formation [45]. Consistent with this mechanism, we found that DcR3 attenuates LPS-induced p38 MAPK phosphorylation in BMDMs. These findings suggest that suppression of p38 activation may contribute, at least in part, to the inhibitory effect of DcR3 on ALIS formation. Notably, p38 MAPK is also a well-established regulator of inflammatory gene expression, including COX-2, TNF-α, and iNOS [65]. However, despite the reduction in p38 activation, DcR3 did not significantly affect LPS-induced expression of COX-2 or iNOS in BMDMs. This apparent discrepancy may be explained by the complex signaling network activated downstream of TLR4. In addition to p38 MAPK, several other pathways, including ERK, JNK, and NF-κB, contribute to the transcriptional regulation of inflammatory mediators. Therefore, the inhibitory effect of DcR3 on p38 signaling may be offset by compensatory regulation through other signaling pathways, resulting in no significant overall change in the expression of COX-2 and iNOS following LPS stimulation. Further studies are required to determine whether DcR3 differentially modulates these signaling pathways and how such regulation contributes to its effects on macrophage function.

In addition to ROS and p38 MAPK signaling, we investigated the potential involvement of HO-1 in the regulation of ALIS formation in DcR3 Tg BMDMs. HO-1 is a stress-inducible enzyme well recognized for its anti-inflammatory, anti-apoptotic, and antioxidant properties. Although LPS stimulation markedly increased HO-1 expression, no significant difference was observed between WT and DcR3 Tg BMDMs. This finding suggests that the inhibitory effect of DcR3 on ROS production and ALIS formation is unlikely to be mediated through altered HO-1 expression. HO-1 is typically induced in response to oxidative stress and inflammatory stimuli, with its expression being regulated primarily by the transcription factor Nrf2. In addition, several other transcription factors, including HIF-1α, NF-κB, and AP-1, have been implicated in the control of HO-1 expression. Multiple signaling pathways, such as MAPKs, PI3K/AKT, and AMPK, also contribute to the regulation of HO-1 transcription and activity [66]. Previous studies have shown that DcR3 knockdown impairs PI3K/AKT signaling in cancer cells [67,68], whereas our current results demonstrate that DcR3 attenuates LPS-induced p38 MAPK activation in BMDMs. Despite these effects on upstream signaling pathways, DcR3 did not alter HO-1 expression following LPS stimulation. One possible explanation is that HO-1 expression is controlled by a highly integrated regulatory network, such that modulation of a single signaling pathway by DcR3 is insufficient to affect the overall level of HO-1 induction. Similar compensatory mechanisms may also account for the lack of effect of DcR3 on the expression of other LPS-responsive genes, including iNOS, COX-2, NLRP3, and pro-IL-1β. Together, these findings suggest that although DcR3 modulates specific signaling events, such as ROS production and p38 MAPK activation, these changes are not necessarily translated into altered expression of downstream inflammatory or stress-response genes due to the redundancy and complexity of the signaling networks activated by LPS.

In addition to assessing HO-1 expression, we examined the effects of pharmacological modulation of HO-1 activity on ALIS formation. Emerging evidence indicates that the heme/HO-1 pathway plays an important role in maintaining cellular proteostasis. Heme is a potent proteotoxic molecule, and excess intracellular heme promotes iron-dependent redox reactions, oxidative protein damage, protein aggregation, and ubiquitination [61], whereas HO-1-mediated heme degradation alleviates proteotoxic stress. Therefore, hemin-induced HO-1 upregulation concomitantly promotes oxidative stress through the generation of ROS and redox-active iron released during heme degradation. Conversely, ZnPP may increase oxidative stress by inhibiting HO-1 activity and thereby impairing the cellular capacity to detoxify excess heme. In addition to inhibiting HO-1 activity, ZnPP might exert HO-1-independent effects. A few studies suggest the effects of ZnPP on inducing protein ubiquitination [69] and altering autophagic functions [70,71], even though the action mechanisms in detail remain unclear. In this study, indeed we found that treatment with hemin modestly increases the formation of ubiquitin-positive puncta, whereas ZnPP markedly promotes the accumulation of ubiquitinated proteins. Interestingly, neither agent further enhances ALIS formation in LPS-stimulated BMDMs, suggesting that their effects are not additive to those induced by LPS. Therefore, the marked induction of ubiquitinated proteins by ZnPP observed in our study may reflect both increased heme-mediated proteotoxic stress and impaired protein degradation. Notably, both hemin and ZnPP have been reported to increase intracellular ROS levels in human colonocytes [72]. Given the established role of ROS in promoting ALIS formation, the effects of hemin and ZnPP on ubiquitinated protein accumulation may be mediated, at least in part, through ROS-dependent mechanisms rather than solely through modulation of HO-1 activity. The ability of DcR3 to suppress ZnPP-induced ubiquitinated protein accumulation further supports a role for DcR3 in the regulation of cellular proteostasis beyond its established anti-inflammatory functions.

In addition to LPS stimulation, we found that DcR3 Tg BMDMs exhibit reduced accumulation of ubiquitinated proteins following treatment with ZnPP, bafilomycin A1, or MG132. These findings suggest that the inhibitory effect of DcR3 on ubiquitinated protein accumulation is not restricted to LPS-induced ALIS formation, but may reflect a broader role in the regulation of cellular protein homeostasis. Protein ubiquitination is a highly coordinated process that depends on the availability of intracellular ubiquitin and the activity of the ubiquitination machinery. In mammals, ubiquitin is encoded by four genes: *UBA52, RPS27A* (formerly *UBA80*), *UBB*, and *UBC*. *UBA52* and *RPS27A* encode ubiquitin fused to ribosomal proteins, whereas *UBB* and *UBC* encode polyubiquitin precursors that are subsequently processed by deubiquitinases to generate ubiquitin monomers. The conjugation of ubiquitin to substrate proteins is mediated sequentially by ubiquitin-activating enzymes (E1), ubiquitin-conjugating enzymes (E2), and ubiquitin ligases (E3), comprising approximately 2, 40, and more than 600 genes, respectively [73,74]. Although the mechanism by which DcR3 reduces ubiquitinated protein accumulation remains unclear, transcriptomic data from the NCBI GEO database provide evidence that DcR3 may influence components of the ubiquitination machinery. For example, ubiquitin-conjugating enzyme E2 J1 (*UBE2J1*) is upregulated in DcR3-treated monocyte-derived macrophages, whereas several E3 ubiquitin ligases, including pellino E3 ubiquitin protein ligase 1 (*PELI1*) and WW domain-containing E3 ubiquitin protein ligase 1 (*WWP1*), are downregulated. These observations raise the possibility that DcR3 may alter the balance between protein ubiquitination and degradation by modulating the expression of ubiquitination-related enzymes. However, because ubiquitinated protein accumulation is determined by both ubiquitination and protein clearance pathways, further studies are needed to determine whether DcR3 directly regulates ubiquitin-encoding genes or other ubiquitination-related factors.

Autophagy plays important roles in both the formation and clearance of ALIS. Because ROS has been identified as an upstream regulator of autophagy [75], and DcR3 attenuated ROS production in BMDMs, we further investigated whether DcR3 influences autophagic activity. Autophagy is initiated by the recruitment of ATG proteins and the nucleation of an isolation membrane, which subsequently elongates and encloses cytoplasmic components to form a double-membraned autophagosome. The autophagosome then fuses with lysosomes to generate an autolysosome, where the sequestered cargo is degraded and recycled [76]. To assess autophagic flux, we utilized bafilomycin A1, an inhibitor of vacuolar H^+^-ATPase that blocks autophagosome–lysosome fusion and lysosomal acidification. Under these conditions, no significant difference in LC3-II accumulation was observed between WT and DcR3 Tg BMDMs. These results suggest that DcR3 does not substantially affect basal autophagic flux, at least as assessed by LC3-II accumulation following bafilomycin A1 treatment. However, because bafilomycin A1 primarily disrupts late-stage autophagic processing, the present data do not exclude the possibility that DcR3 influences other aspects of autophagy, such as autophagosome formation, cargo recognition, selective autophagy, or lysosomal function. Therefore, further studies are required to clarify the precise role of DcR3 in the regulation of autophagy and lysosome-dependent protein quality-control pathways.

Because DcR3 is naturally expressed in humans but absent from the mouse genome, validation of our findings in human macrophages is essential to establish their physiological and clinical relevance. Therefore, it is necessary for future studies to investigate the role of endogenous DcR3 in ALIS responses in human macrophages. Such studies will help determine whether the inhibitory effects of DcR3 on ALIS formation, ubiquitinated protein accumulation, and proteostasis observed in DcR3 transgenic mouse macrophages can be recapitulated in human cells. Furthermore, investigating the role of endogenous DcR3 in human macrophages may provide additional insights into how DcR3 regulates innate immune responses, protein quality-control pathways, and stress adaptation under inflammatory conditions.

In summary, our findings demonstrate that DcR3 does not significantly affect the expression of major inflammatory mediators in response to LPS stimulation in BMDMs. Nevertheless, DcR3 markedly suppresses LPS-induced ALIS formation, an effect that is associated with reduced cellular ROS production and attenuated p38 MAPK activation. We further show that autophagy negatively regulates ALIS formation and that DcR3 decreases the accumulation of ubiquitinated proteins induced not only by LPS, but also by ZnPP, bafilomycin A1, and MG132. Collectively, our study identifies a previously unrecognized function of DcR3 in the regulation of ALIS dynamics and ubiquitinated protein accumulation in macrophages. Given the established roles of ALIS in protein quality control, stress adaptation, and antigen presentation, the ability of DcR3 to limit ALIS formation may represent an important mechanism for preserving cellular homeostasis during innate immune activation. Further studies are warranted to elucidate the molecular basis by which DcR3 regulates ROS homeostasis, p38 MAPK signaling, autophagy, and protein ubiquitination, and to determine how these pathways collectively contribute to the control of ALIS formation and proteostasis in macrophages.

## 4. Materials and Methods

### 4.1. Reagents and Antibodies

DMEM, FBS, and trypsin-EDTA were purchased from Gibco (Carlsbad, CA, USA). Penicillin-streptomycin solution was obtained from Biological Industries (Kibbutz Beit-Haemek, Israel). Dulbecco’s PBS, LPS, N-acetyl-L-cysteine (NAC), hemin, bafilomycin A1, MG132, rapamycin, and protease inhibitor cocktails were purchased from Sigma-Aldrich (St. Louis, MO, USA). SB203580 was purchased from Calbiochem (San Diego, CA, USA). Zinc protoporphyrin (ZnPP) was bought from MedChemExpress (Monmouth Junction, NJ, USA). Antibodies against COX-2 (#12282), SQSTM1/p62 (#5114), phospho-p38 (Thr180/Tyr182) (#9211), horseradish peroxidase (HRP)-linked anti-mouse IgG (#7076), HRP-linked anti-rabbit IgG (#7074), Alexa Fluor 555 conjugated anti-mouse IgG (#4409), and Alexa Fluor 488 conjugated anti-rabbit IgG (#4412) were obtained from Cell Signaling Technology (Danvers, MA, USA). Antibodies of β-actin (sc-47778), ubiquitin (sc-8017), and HRP-linked donkey anti-goat IgG (sc-2033) were purchased from Santa Cruz Biotechnology (Dallas, TX, USA). Antibody of DcR3 (333202) was obtained from BioLegend (San Diego, CA, USA). Antibody of NLRP3/NALP3 (AG-20B-0014) was from AdipoGen (San Diego, CA, USA). Antibody against IL-1β (AF-401-NA) was purchased from R&D Systems (Minneapolis, MN, USA). Antibody against iNOS (ab178945), K48 (ab140601), and K63 (ab179434) linkage-specific Ubiquitin were bought from Abcam (Cambridge, UK). Antibody of LC3 (PM-036) was purchased from MBL International (Woburn, MA, USA). Antibody against HO-1 (ADI-SPA-896) was obtained from Enzo Life Science (Farmingdale, NY, USA).

### 4.2. Mice and Ethics Statement

CD68 promoter-driven DcR3 Tg mice expressing DcR3 in myeloid cells were generated as previously described [77]. In brief, human *DcR3* cDNA was placed under the control of the human *CD68* promoter to direct transgene expression predominantly in myeloid cells. The transgene construct was introduced into fertilized mouse embryos by pronuclear microinjection, and transgenic founder mice were identified by PCR genotyping and bred to establish stable DcR3 Tg lines. Because mice lack an endogenous *DcR3* gene, this model allows selective expression of human DcR3 in CD68-positive myeloid cells. We selected mice with a high titer of DcR3 level (>1 μg/mL) by serum ELISA for the experiments. DcR3 expression was confirmed by immunoblotting. All mice (C57BL/6 background) were bred in specific-pathogen-free conditions at the National Taiwan University College of Medicine Laboratory Animal Center. The mouse experimental protocols were reviewed and approved by the Institutional Animal Care and Use Committee (IACUC 20201019). The mouse experiments were performed in accordance with the recommendations in the Guideline for the Care and Use of Laboratory Animals of IACUC, National Taiwan University College of Medicine, and College of Public Health.

### 4.3. Enzyme-Linked Immunosorbent Assay (ELISA) of DcR3

DuoSet ELISA kit (DY142, R&D Systems, Minneapolis, MN, USA) was used for the determination of DcR3 level in mouse serum following the manufacturer’s instructions. For plate preparation, a 96-well plate was coated with capture antibody and incubated at room temperature overnight. The plate was blocked with reagent diluent (1% bovine serum albumin) in PBS for 2 h. For the assay procedure, serum samples (500× dilution in reagent diluent) were added and incubated for 2 h. Detection antibody was added and incubated for 2 h. Streptavidin-HRP was added and incubated for 20 min in the dark. Substrate solution was added and incubated for 20 min in the dark. 2 N H_2_SO_4_ was added as a stop solution and incubated in the dark for an appropriate time depending on color development. Except for the stopping step, each well was aspirated and subjected to three washes with wash buffer (0.05% Tween-20 in PBS) at every step. All procedures were performed at room temperature.

### 4.4. Cell Culture of Bone Marrow-Derived Macrophages

BMDMs were obtained from 8- to 12-week-old WT and DcR3 Tg mice. Mice were sacrificed by cervical dislocation. Tibias and femurs were dissected, and the adhering tissue was removed. Both ends of the bones were cut off and flushed with DMEM through 25-gauge needles until the bones became white. The cells were suspended by vigorous pipetting, and the suspension was passed through a 70 μm nylon strainer to remove the remaining bones and tissue. The suspension was centrifuged at 1000 rpm for 10 min. After removal of supernatant, the cell pellet was suspended in DMEM and cultured in three 150 mm Petri dishes with each 20 mL DMEM supplemented with 10% conditioned medium of L929 fibroblasts, 10% FBS, and 1% penicillin/streptomycin. After 5 days of incubation, BMDMs were obtained from the adherent cells. Conditioned medium of L929 fibroblasts was collected from the culture medium of L929 fibroblasts, which were cultured at 5 × 10^5^ cells for 7 to 10 days with 25 mL DMEM in a 75T flask. Cells were incubated at 37 °C in a humidified atmosphere of 5% CO_2_ and 95% air.

### 4.5. Immunofluorescence

BMDMs (3 × 10^5^ cells/well) were seeded on cover glasses in a 12-well plate. After different treatments, the media were removed. Cells were rinsed with PBS and fixed with 4% paraformaldehyde (Sigma-Aldrich, St. Louis, MO, USA) for 15 min at room temperature. After washing three times with PBS, cells were permeabilized with 0.2% Triton X-100 in PBS for 20 min at room temperature. Cells were then blocked with 4% BSA for 1 h and incubated with primary antibodies against ubiquitin, p62, and LC3 for 2 h at room temperature or overnight at 4 °C after aspiration of the blocking solution. Later, the primary antibodies were removed, and the cells were washed three times with PBS. Cover glasses were incubated with fluorochrome-conjugated secondary antibody in the dark at room temperature. Following immunostaining, cover glasses were counterstained with 4′,6-DAPI (SouthernBiotech, Birmingham, AL, USA) and mounted on the microscope slide in the dark. Images were performed by LSM 780 confocal microscope (Zeiss, Oberkochen, Germany). 

### 4.6. Quantification of ALIS Formation

Cells were immunostained with antibodies against ubiquitin, p62, or LC3 and examined by fluorescence microscopy. Images were acquired under identical exposure settings for all experimental groups. For each condition, 4–6 randomly selected microscopic fields containing approximately 3–10 cells per field were analyzed in each independent experiment. The percentage of cells containing ubiquitin- or p62-positive puncta and the average number of ubiquitin-, p62-, or LC3-positive puncta per cell were quantified using ImageJ software (version 1.54). Based on the colocalization of ubiquitin and p62, ubiquitin-positive puncta were subsequently used as a surrogate marker for ALIS. ALIS formation was quantified by determining the percentage of ALIS-positive cells (cells containing ubiquitin-positive puncta) and the number of ALIS puncta per cell. Data were obtained from three independent experiments (*n* = 3) and are presented as the mean ± SEM.

### 4.7. Immunoblotting

BMDMs (5 × 10^5^ cells) were seeded in a 35 mm Petri dish with DMEM supplemented with 10% FBS and 1% penicillin/streptomycin. After different treatments, media were removed, and cells were lysed with radioimmunoprecipitation assay (RIPA) buffer (50 mM Tris-HCl, pH 7.4; 150 mM NaCl; 0.1% sodium dodecyl sulfate (SDS); 0.1% sodium deoxycholate; 1 mM phenylmethylsulfonyl fluoride; 2 mM EDTA; 2 mM NaF; 2 mM Na_3_VO_4_; 1% Triton X-100; 0.2% protease inhibitor cocktail). After harvest and sonication, the protein concentration of total cell lysates was determined by Bio-Rad protein assay dye reagent (Bio-Rad, Hercules, CA, USA). Equal amounts of protein (20 μg) were subjected to 8-15% SDS-PAGE and transferred to Amersham Hybond P 0.45 μm PVDF membranes (Cytiva, Marlborough, MA, USA). Membranes were blocked with 5% (*w*/*v*) skimmed milk in Tris-buffered saline with Tween 20 (TBST, 50 mM Tris-HCl, pH 7.4; 150 mM NaCl; 0.1% Tween 20) for 1 h at room temperature. After rinsing with TBST, membranes were incubated with primary antibody at 4 °C overnight. Later, membranes were washed with TBST (10 min × 3 times) and incubated with HRP-linked secondary antibody for 1 h at room temperature. After incubation, membranes were washed with TBST (10 min × 3 times), and protein bands were detected with enhanced chemiluminescence reagents (PerkinElmer, Waltham, MA, USA; Merck Millipore, Burlington, MA, USA) by ChemiDoc MP Imaging System (Bio-Rad, Hercules, CA, USA). β-Actin was used as an internal control. Quantitative analyses were performed by Image Lab software (version 6.1).

### 4.8. Measurement of Cellular Reactive Oxygen Species

Cellular reactive oxygen species (ROS) were measured by H_2_DCFDA (Invitrogen, Carlsbad, CA, USA). BMDMs (5 × 10^5^ cells) were seeded in a 35 mm Petri dish. After treatments, cells were detached from the dish by trypsin-EDTA, collected, and centrifuged at 2000 rpm for 5 min. After removal of supernatants, cell pellets were suspended in PBS with H_2_DCFDA (10 μM) followed by incubation at 37 °C for 30 min. Later, the suspension was centrifuged at 2000 rpm for 5 min. After removal of supernatant, cell pellets were resuspended in PBS. The fluorescence intensity was detected by FACSCalibur flow cytometry (BD, Franklin Lakes, NJ, USA). Analyses were performed using CellQuest Pro software, (version 5.1) and relative protein expression was compared after normalization with β-actin.

### 4.9. Statistical Analysis

The experimental values were presented as mean ± standard error of the mean (S.E.M) from three independent experiments (*n* = 3). Student’s *t*-test was used to determine the statistical significance of the differences (*p* < 0.05). Statistical analyses were performed by GraphPad Prism 8 software.

## Data Availability

Data will be made available on request.

## Abbreviations

The following abbreviations are used in this manuscript:

ALIS	Aggresome-like induced structures
BMDMs	Bone marrow-derived macrophages
CIITA	Class II transactivator
CLP	Cecal ligation and puncture
CRDs	Cysteine-rich domains
DcR3	Decoy receptor 3
DcR3.Fc	DcR3-Fc fusion protein
DcR3 Tg	DcR3 transgenic
DR3	Death receptor 3
ELISA	Enzyme-linked immunosorbent assay
FasL	Fas ligand
HBD	HSPG binding domain
HO-1	Heme oxygenase
HSPG	Heparan sulfate proteoglycan
HVEM	Herpesvirus entry mediator
LPS	Lipopolysaccharide
LTβR	Lymphotoxin β receptor
MHC	Major histocompatibility complex
MHC-1	MHC class I
NAC	N-acetyl-L-cysteine
NOXs	NADPH oxidases
ROS	Reactive oxygen species
TNFR	Tumor necrosis factor receptor
WT	Wild-type
ZnPP	Zinc protoporphyrin
